# Metabolomics profile and 10-year atherosclerotic cardiovascular disease (ASCVD) risk score

**DOI:** 10.3389/fcvm.2023.1161761

**Published:** 2023-05-03

**Authors:** Hojat Dehghanbanadaki, Salimeh Dodangeh, Peyvand Parhizkar Roudsari, Shaghayegh Hosseinkhani, Pouria Khashayar, Mohammad Noorchenarboo, Negar Rezaei, Arezou Dilmaghani-Marand, Moein Yoosefi, Babak Arjmand, Kazem Khalagi, Niloufar Najjar, Ardeshir Kakaei, Fatemeh Bandarian, Hamid Aghaei Meybodi, Bagher Larijani, Farideh Razi

**Affiliations:** ^1^ Endocrinology and Metabolism Research Center, Endocrinology and Metabolism Clinical Sciences Institute, Tehran University of Medical Sciences, Tehran, Iran; ^2^ Diabetes Research Center, Endocrinology and Metabolism Clinical Sciences Institute, Tehran University of Medical Sciences, Tehran, Iran; ^3^ Metabolic Disorders Research Center, Endocrinology and Metabolism Molecular—Cellular Sciences Institute, Tehran University of Medical Sciences, Tehran, Iran; ^4^ Institute of Cardiovascular and Medical Sciences, University of Glasgow, Glasgow, United Kingdom; ^5^ Department of Epidemiology and Biostatistics, School of Public Health, Tehran University of Medical Sciences, Tehran, Iran; ^6^ Non-Communicable Diseases Research Center, Endocrinology and Metabolism Population Sciences Institute, Tehran University of Medical Sciences, Tehran, Iran; ^7^ Cell Therapy and Regenerative Medicine Research Center, Endocrinology and Metabolism Molecular-Cellular Sciences Institute, Tehran, Iran; ^8^ Obesity and Eating Habits Research Center, Endocrinology and Metabolism Clinical Sciences Institute, Tehran University of Medical Sciences, Tehran, Iran; ^9^ Osteoporosis Research Center, Endocrinology and Metabolism Clinical Sciences Institute, Tehran University of Medical Sciences, Tehran, Iran; ^10^ Metabolomics and Genomics Research Center, Endocrinology and Metabolism Molecular-Cellular Sciences Institute, Tehran University of Medical Sciences, Tehran, Iran; ^11^ Personalized Medicine Research 10-Center, Endocrinology and Metabolism Clinical Sciences Institute, Tehran University of Medical Sciences, Tehran, Iran

**Keywords:** cardiovascular disease, ASCVD, metabolomics, amino acids, acylcarnitine

## Abstract

**Background:**

The intermediate metabolites associated with the development of atherosclerotic cardiovascular disease (ASCVD) remain largely unknown. Thus, we conducted a large panel of metabolomics profiling to identify the new candidate metabolites that were associated with 10-year ASCVD risk.

**Methods:**

Thirty acylcarnitines and twenty amino acids were measured in the fasting plasma of 1,102 randomly selected individuals using a targeted FIA-MS/MS approach. The 10-year ASCVD risk score was calculated based on 2013 ACC/AHA guidelines. Accordingly, the subjects were stratified into four groups: low-risk (*n* = 620), borderline-risk (*n* = 110), intermediate-risk (*n* = 225), and high-risk (*n* = 147). 10 factors comprising collinear metabolites were extracted from principal component analysis.

**Results:**

C_4_DC, C_8:1_, C_16_OH, citrulline, histidine, alanine, threonine, glycine, glutamine, tryptophan, phenylalanine, glutamic acid, arginine, and aspartic acid were significantly associated with the 10-year ASCVD risk score (*p*-values ≤ 0.044). The high-risk group had higher odds of factor 1 (12 long-chain acylcarnitines, OR = 1.103), factor 2 (5 medium-chain acylcarnitines, OR = 1.063), factor 3 (methionine, leucine, valine, tryptophan, tyrosine, phenylalanine, OR = 1.074), factor 5 (6 short-chain acylcarnitines, OR = 1.205), factor 6 (5 short-chain acylcarnitines, OR = 1.229), factor 7 (alanine, proline, OR = 1.343), factor 8 (C_18:2_OH, glutamic acid, aspartic acid, OR = 1.188), and factor 10 (ornithine, citrulline, OR = 1.570) compared to the low-risk ones; the odds of factor 9 (glycine, serine, threonine, OR = 0.741), however, were lower in the high-risk group. “D-glutamine and D-glutamate metabolism”, “phenylalanine, tyrosine, and tryptophan biosynthesis”, and “valine, leucine, and isoleucine biosynthesis” were metabolic pathways having the highest association with borderline/intermediate/high ASCVD events, respectively.

**Conclusions:**

Abundant metabolites were found to be associated with ASCVD events in this study. Utilization of this metabolic panel could be a promising strategy for early detection and prevention of ASCVD events.

## Introduction

Cardiovascular disease (CVD) is a disorder of the heart/vessels known as the most important reason behind global mortality. The contribution of CVD to the death rate has increased continuously (1, 2). Therefore, it is crucial to identify high-risk individuals to prevent CVD and its associated complications and reduce the burden of the disease and mortality (3). To this purpose, different CVD risk assessment scoring systems have been developed so far (4). One of the recent most-applied tools is the 10-year atherosclerotic CVD (ASCVD) risk index that was presented by the American Heart Association (AHA)/ the American College of Cardiology (ACC) (5). This risk score was calculated based on several characteristics of individuals including age, sex, race, total cholesterol, high-density lipoprotein cholesterol (HDL-C), systolic blood pressure, antihypertensive treatment, smoking status, and diabetes mellitus history (6, 7).

Although there have been many advances in the development of predictive CVD risk assessment tools, the underlying molecular pathomechanisms of ASCVD events are largely unrecognized. This issue revealed the necessity for conducting metabolomics analysis (8). The metabolomics approach allows for a better understanding of the intermediate metabolites associated with ASCVD events and leads to the identification of new diagnostic and therapeutic strategies (9, 10). Indeed, physiologic perturbations, particularly in individuals at high risk of ASCVD events, can rapidly affect metabolite profiling, which can be targeted for disease management (11). Herein, several metabolomics studies have been conducted to determine the association between metabolic profiles and CVD risk, which showed that branched-chain amino acids and urea cycle-related metabolites, were associated with higher cardiovascular risk (12, 13). However, these studies identified the association between a number of acylcarnitines/amino acids and CVD risk, but the results are not easily comparable, e.g., isoleucine, leucine, and glutamine were associated with cardiovascular events in one study (13) while these metabolites were not associated with an increased incidence of cardiovascular events in another study (12). So, intermediate metabolites associated with developing ASCVD events remain largely unknown. Thus, to better understand the pathogenesis of the disease, in this study, we conducted the comprehensive metabolomics profiling of plasma in a large-scale Iranian population and determined the association between plasma metabolite levels and the 10-year ASCVD risk score.

## Methods

### Study subjects

1,102 individuals aged between 40 and 79 years old with LDL levels less than 190 mg/dl and no pre-existing coronary artery disease or myocardial infarction were randomly selected from a survey of Surveillance of Risk Factors of Non-Communicable Diseases in Iran (STEPs 2016). In brief, the STEPs 2016 protocol, previously published, includes 31,050 individuals older than 18 years from the rural and urban areas of 389 Iranian districts selected using a systematic cluster random sampling (14). The study protocol conforms to the ethical guidelines of the 1975 Declaration of Helsinki. The Ethics Committee of Tehran University of Medical Sciences and Endocrine & Metabolism Research Institute approved the study protocol with the ID number IR.TUMS.EMRI.REC.1395.00141, and written informed consent was obtained before participation.

### Blood sampling and laboratory assessment

The blood was sampled from peripheral venous following at least 12 h of overnight fasting and stored in sodium fluoride plus EDTA tubes. A part of the whole blood was used for HbA1c measurement and the plasma of the remaining blood was isolated for other biochemical laboratory testing using commercial Roche kits (Roche Diagnostics, Mannheim, Germany) and Cobas C311 auto analyzer.

### Plasma metabolic profiling

The concentration of 30 acylcarnitines and 20 amino acids were measured in plasma using flow injection tandem mass spectrometry (triple quadrupole SCIEX API 3,200 with electrospray ionization) equipped with a Thermo Scientific Dionex UltiMate 3,000 standard HPLC system and a derivatization method with butanol-HCL (15). Briefly, the mixture of plasma samples and internal standard were centrifuged at 4°C. Supernatant fluids were collected in new vials and dried with nitrogen 99.9% at 45°C. Derivatization solution, a combination of 1-butanol and acetyl chloride, was then added to the vials with the aim of protein precipitation. Also, derivatization with butanol-HCL has the benefit of simultaneous derivatization of amino acids and acylcarnitines which makes the measurement, faster and cheaper. After vortexing, they were incubated at 65°C for 15 min with the advantage of the effective destruction of phospholipids. The samples were dried with nitrogen 99.9% at 45°C, then dissolved in 100 µl of mobile phase solution, a mix of water and acetonitrile, before injection. Ratios of the signals of the metabolites relative to the internal standards were used to make calibration curves and calculate analyte concentrations in the QC materials and samples.

### 10-year ASCVD risk score assessment

The 10-year risk for primary atherosclerotic cardiovascular disease was calculated based on pooled cohort equations in the 2013 ACC/AHA Guidelines (16). The equation predicts the risk of stroke, nonfatal myocardial infarction, and coronary artery disease death within 10 years in subjects aged between 40 and 79 years old with LDL < 190 mg/dl and no pre-existing ASCVD events. The equation takes into account age, gender, race, HDL cholesterol, total cholesterol, systolic blood pressure, the use of hypertension drugs, smoking habits, and diabetes. The individuals were stratified into four groups according to their 10-year ASCVD risk score and the threshold was determined based on the special report from AHA/ACC (6): (1) Low-risk group (*n* = 620) with a score of 0% to 5%, (2) Borderline-risk group (*n* = 110) with a score of 5% to 7.5%, (3) Intermediate-risk group (*n* = 225) with a score of 7.5% to 20%, and (4) High-risk group (*n* = 147) with a score of 20% or more.

### Statistical analysis

SPSS version 19.0 was used for statistical analyses. The workflow diagram of the statistical analysis is presented in Additional File S1. After checking the normality by Kolmogorov–Smirnov test, mean (± standard deviation) and median (interquartile range) were applied to describe continuous variables with and without normal distributions, respectively. Number and percentage (%) were applied to describe categorical variables. Chi-squared test was used to compare the frequency of categorical variables between the groups. ANOVA test and Bonferroni *post hoc* test were used to compare normally distributed variables between the groups. The Kruskal-Wallis and Mann–Whitney U tests were used to compare non-normally distributed variables between the groups. Benjamini-Hochberg method was applied for the adjustment of *p*-values obtained from the Kruskal-Wallis H test of the metabolite concentrations. The correlation between the metabolite concentrations and the 10-year ASCVD risk was determined using the Spearman correlation coefficient and the stepwise multiple linear regression analysis methods. To standardize the data on metabolite level, we calculated the natural logarithm of metabolite concentration and considered their *Z* value for logistic regression analysis and factor analysis. Binary logistic regression analysis was used to determine the odds ratio (OR) of metabolite profile in the borderline/intermediate/high-risk groups compared to the low-risk one. For factor analysis, the Kaiser-Meyer-Olkin (KMO) test and Bartlett's Test of Sphericity were used to investigate the adequacy of sample size and for statistical comparison of the correlation matrix with the identity matrix. KMO values ≥ 0.80 were considered credible. Principal component analysis (PCA) with varimax rotation was performed to reduce the metabolites into a smaller subclass of orthogonal (uncorrelated) factors. The extracted factors with eigenvalues higher than 1.0 and metabolites with loading scores more than 0.4 were considered important for the given PCA. The factor score was calculated through the sum of obtained metabolite concentrations multiplied by their loading matrix which was obtained from the rotated component matrix with the rotation method of Varimax with Kaiser Normalization (Additional File S2). Furthermore, MetaboAnalyst (Version 5.0) based on the metabolic Kyoto Encyclopedia of Genes and Genomes (KEGG) database was used for enrichment pathway analysis of metabolic alterations between study groups that were identified from logistic regression analysis. The enrichment ratio is calculated based on the observed hits divided by expected hits. A *p*-value less than 0.05 was considered significant in all analyses.

## Results

### General characteristics of the study population

The study population comprised 1,102 participants with a mean age of 54.41 ± 10.22 years and a female percentage of 53.27%. The sociodemographic and laboratory characteristics of the low/borderline/intermediate/high ASCVD risk groups are shown in Table 1. The groups had no significant difference with regard to BMI and cholesterol levels (*p*-value ≥ 0.056).

**Table 1:** The general characteristics of participants stratified by 10-year ASCVD risk score.

Variables		Low-risk (*n* = 620)	Borderline-risk (*n* = 110)	Intermediate-risk (*n* = 225)	High-risk (*n* = 147)	*p*-value
Age (year)		48.32 ± 5.98	55.15 ± 7.05	60.34 ± 7.33	70.53 ± 6.64	**<0**.**001**
Female, *n* (%)		425 (69)	47 (43)	72 (32)	43 (29)	**<0**.**001**
BMI (kg/m^2^)		28.24 ± 5.32	28.17 ± 4.97	27.15 ± 4.73	27.50 ± 4.81	0.056
WC (cm)		93.92 ± 13.27	96.17 ± 13.00	96.11 ± 12.89	98.03 ± 13.02	**<0**.**001**
HC (cm)		103.54 ± 11.59	103.27 ± 10.84	101.25 ± 9.79	101.13 ± 10.82	**0**.**002**
WC/HC		0.91 ± 0.09	0.93 ± 0.09	0.95 ± 0.08	0.97 ± 0.10	**<0**.**001**
SBP (mm Hg)		124.44 ± 16.01	131.25 ± 18.53	138.80 ± 21.37	151.34 ± 20.31	**<0**.**001**
DBP (mm Hg)		78.79 ± 10.63	80.19 ± 11.20	84.21 ± 12.63	84.79 ± 13.33	**<0**.**001**
HDL-C (mg/dl)		42.91 ± 11.31	39.51 ± 10.88	38.82 ± 11.37	39.16 ± 11.56	**<0**.**001**
TG (mg/dl)		126.19 ± 74.98	147.81 ± 85.08	166.96 ± 158.20	138.94 ± 79.30	**<0**.**001**
Cholesterol (mg/dl)		167.24 ± 33.65	169.13 ± 33.84	171.78 ± 38.93	167.92 ± 38.95	0.414
Non-HDL-C (mg/dl)		124.34 ± 33.22	129.62 ± 34.27	132.96 ± 39.57	128.76 ± 36.12	**0**.**025**
HbA1c (%)		5.56 ± 0.71	5.93 ± 1.26	6.15 ± 1.35	6.57 ± 1.53	**<0**.**001**
FPG (mg/dl)		95.80 ± 23.37	109.16 ± 53.20	110.13 ± 44.08	119.07 ± 51.05	**<0**.**001**
10-year ASCVD risk		2.05 ± 1.26	6.11 ± 0.68	12.70 ± 3.65	33.67 ± 12.09	**<0**.**001**
Area of living, *n* (%)	Rural	201 (32)	36 (33)	85 (38)	45 (31)	**<0**.**001**
	Urban	419 (68)	74 (67)	140 (62)	102 (69)	
Education (years), *n* (%)	<1	113 (18)	23 (21)	77 (34)	57 (39)	**<0**.**001**
	1 to 6	216 (35)	39 (35)	74 (33)	51 (35)	
	7 to 12	215 (35)	37 (34)	46 (20)	17 (12)	
	>12	76 (12)	11 (10)	28 (12)	22 (15)	
Smoking, *n* (%)		46 (7)	21 (19)	61 (27)	31 (21)	**<0**.**001**
Diabetes, *n* (%)		36 (6)	22 (20)	52 (23)	71 (48)	**<0**.**001**

BMI, body mass index; WC, waist circumference; HC, hip circumference; SBP, systolic blood pressure; DBP, diastolic blood pressure; HDL-C, high density lipoprotein cholesterol; TG, triglyceride; Non-HDL-C, non-high-density lipoprotein cholesterol; HbA1c, hemoglobin A1c; FPG, fasting plasma glucose.

### Metabolite profile as a 10-year ASCVD risk score predictor

**Figure 1:**
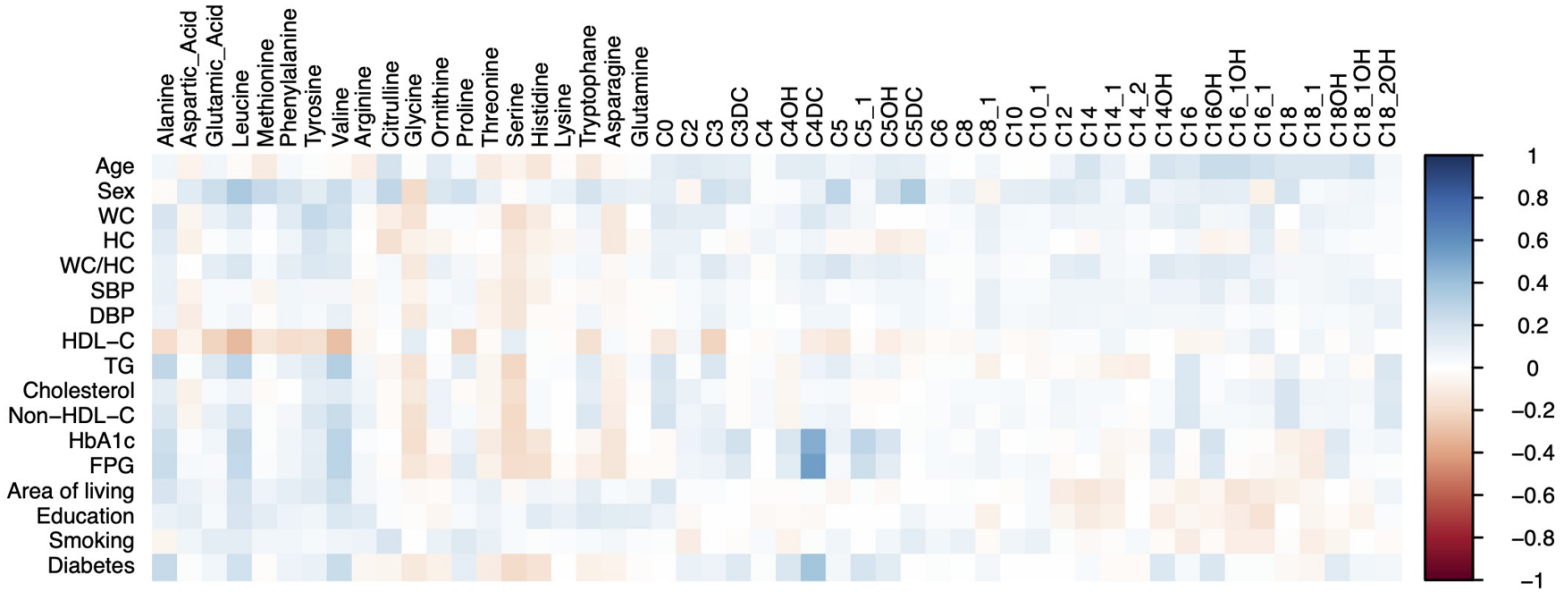
Correlation matrix between metabolites and general features of participants.

Tables 2, 3 demonstrate the median (interquartile range) of plasma concentration of 30 acylcarnitines and 20 amino acids in the four study groups, respectively. For ease of visualization of the data scatter, the concentration of acylcarnitines/amino acids in the four study groups is plotted in Additional File S3. The correlation matrix between metabolites and general features of participants is shown in Figure 1. The significantly altered metabolites between male and female participants for the four study groups are shown in Additional File S4.

**Table 2:** The plasma concentration for acylcarnitines stratified by 10-year ASCVD risk score.

Acylcarnitines (μmol/l)	Low-risk	Borderline-risk	Intermediate-risk	High-risk	FDR*
C_0_	54.251 (45.854–61.755)	56.876 (48.720–66.211)	58.995 (50.103–66.252)	55.313 (47.701–66.281)	**<0**.**001**
C_2_	13.547 (11.317–16.249)	14.232 (11.583–16.534)	13.665 (11.762–16.310)	14.752 (12.163–17.516)	**0**.**014**
C_3_	0.734 (0.571–0.955)	0.856 (0.666–1.180)	0.878 (0.695–1.117)	0.934 (0.697–1.172)	**<0**.**001**
C_3_DC	0.072 (0.055–0.099)	0.079 (0.063–0.101)	0.080 (0.057–0.106)	0.090 (0.067–0.117)	**<0**.**001**
C_4_	0.363 (0.286–0.490)	0.393 (0.307–0.556)	0.412 (0.334–0.515)	0.437 (0.346–0.536)	**<0**.**001**
C_4_OH	0.049 (0.038–0.065)	0.053 (0.043–0.065)	0.052 (0.040–0.067)	0.057 (0.045–0.075)	**<0**.**001**
C_4_DC	0.062 (0.048–0.078)	0.070 (0.057–0.084)	0.071 (0.058–0.087)	0.075 (0.059–0.101)	**<0**.**001**
C_5_	0.197 (0.155–0.256)	0.221 (0.174–0.269)	0.239 (0.187–0.304)	0.237 (0.192–0.296)	**<0**.**001**
C_5:1_	0.035 (0.027–0.052)	0.040 (0.029–0.052)	0.037 (0.030–0.054)	0.041 (0.031–0.058)	**0**.**002**
C_5_OH	0.060 (0.051–0.072)	0.068 (0.056–0.078)	0.066 (0.057–0.077)	0.069 (0.061–0.079)	**<0**.**001**
C_5_DC	0.283 (0.229–0.366)	0.318 (0.266–0.386)	0.325 (0.253–0.401)	0.351 (0.277–0.449)	**<0**.**001**
C_6_	0.164 (0.120–0.239)	0.169 (0.129–0.230)	0.170 (0.123–0.242)	0.183 (0.136–0.260)	0.140
C_8_	0.245 (0.167–0.353)	0.253 (0.188–0.372)	0.251 (0.175–0.370)	0.275 (0.192–0.415)	0.080
C_8:1_	0.284 (0.199–0.408)	0.329 (0.258–0.458)	0.290 (0.216–0.416)	0.322 (0.225–0.408)	**0**.**009**
C_10_	0.315 (0.215–0.481)	0.331 (0.245–0.491)	0.323 (0.212–0.483)	0.358 (0.242–0.567)	0.057
C_10:1_	0.320 (0.230–0.441)	0.340 (0.257–0.453)	0.315 (0.224–0.458)	0.340 (0.251–0.502)	0.150
C_12_	0.120 (0.092–0.173)	0.135 (0.101–0.172)	0.137 (0.104–0.183)	0.151 (0.104–0.207)	**<0**.**001**
C_14_	0.049 (0.039–0.064)	0.055 (0.043–0.068)	0.056 (0.045–0.073)	0.061 (0.045–0.081)	**<0**.**001**
C_14:1_	0.109 (0.081–0.162)	0.116 (0.090–0.158)	0.111 (0.082–0.167)	0.125 (0.088–0.187)	0.080
C_14:2_	0.085 (0.063–0.116)	0.086 (0.071–0.115)	0.088 (0.065–0.121)	0.096 (0.072–0.135)	0.161
C_14_OH	0.011 (0.008–0.014)	0.012 (0.009–0.015)	0.013 (0.010–0.016)	0.014 (0.010–0.018)	**<0**.**001**
C_16_	0.168 (0.142–0.203)	0.175 (0.157–0.214)	0.184 (0.152–0.224)	0.189 (0.160–0.231)	**<0**.**001**
C_16_OH	0.010 (0.008–0.012)	0.011 (0.008–0.013)	0.012 (0.010–0.014)	0.013 (0.010–0.016)	**<0**.**001**
C_16:1_OH	0.015 (0.012–0.020)	0.016 (0.013–0.021)	0.017 (0.013–0.024)	0.018 (0.014–0.026)	**<0**.**001**
C_16:1_	0.042 (0.032–0.058)	0.044 (0.033–0.056)	0.044 (0.034–0.061)	0.050 (0.036–0.068)	**0**.**005**
C_18_	0.060 (0.049–0.073)	0.065 (0.052–0.077)	0.067 (0.056–0.086)	0.068 (0.056–0.084)	**<0**.**001**
C_18:1_	0.169 (0.135–0.218)	0.177 (0.138–0.222)	0.177 (0.143–0.231)	0.174 (0.145–0.230)	0.068
C_18_OH	0.008 (0.006–0.010)	0.008 (0.007–0.010)	0.009 (0.007–0.011)	0.010 (0.008–0.012)	**<0**.**001**
C_18:1_OH	0.011 (0.009–0.014)	0.012 (0.010–0.015)	0.012 (0.010–0.016)	0.013 (0.010–0.017)	**<0**.**001**
C_18:2_OH	0.027 (0.021–0.035)	0.029 (0.022–0.037)	0.029 (0.022–0.038)	0.029 (0.024–0.039)	**0**.**015**

*FDR: False Discovery Rate (Adjusted P-value).

**Table 3:** The plasma concentration for amino acids stratified by a 10-year ASCVD risk score.

Amino acids (μmol/l)	Low-risk	Borderline-risk	Intermediate-risk	High-risk	FDR*
Alanine	401.30 (337.65–471.10)	426.40 (363.60–494.50)	426.50 (374.40–479.20)	423.00 (373.20–499.10)	**<0**.**001**
Aspartic acid	12.45 (10.10–14.75)	12.65 (10.40–15.00)	12.50 (10.60–14.70)	12.40 (10.00–14.60)	0.844
Glutamic acid	65.50 (57.90–72.20)	68.55 (62.50–75.20)	68.80 (61.90–77.20)	67.30 (61.90–76.00)	**<0**.**001**
Leucine	118.65 (102.85–134.40)	131.95 (109.50–146.60)	130.20 (115.40–150.70)	126.40 (115.30–147.30)	**<0**.**001**
Methionine	27.50 (24.60–31.70)	28.40 (24.50–31.90)	27.80 (24.80–31.90)	27.60 (24.40–30.50)	0.455
Phenylalanine	61.85 (55.00–69.20)	62.30 (55.30–69.30)	66.30 (59.20–73.50)	67.40 (60.20–73.60)	**<0**.**001**
Tyrosine	68.70 (59.80–79.10)	71.75 (65.10–80.30)	70.70 (63.90–82.00)	70.80 (63.30–80.50)	**0**.**005**
Valine	249.75 (220.95–282.30)	266.80 (236.50–303.60)	274.70 (239.00–310.10)	269.50 (237.80–299.20)	**<0**.**001**
Arginine	67.95 (56.00–83.65)	72.00 (60.10–82.30)	70.30 (54.40–83.50)	68.10 (55.50–80.70)	0.694
Citrulline	35.65 (30.20–40.70)	39.85 (34.50–45.20)	39.60 (33.20–45.10)	40.80 (35.30–48.20)	**<0**.**001**
Glycine	256.80 (211.0–327.55)	253.25 (222.60–298.00)	233.90 (203.10–284.00)	240.30 (206.40–293.60)	**0**.**003**
Ornithine	85.70 (73.20–100.00)	88.30 (76.00–105.60)	93.00 (81.30–111.50)	89.50 (76.70–104.40)	**<0**.**001**
Proline	230.30 (191.50–278.60)	253.75 (206.90–305.60)	257.60 (208.50–310.10)	251.00 (206.50–307.30)	**<0**.**001**
Threonine	138.24 (116.70–158.40)	136.45 (117.90–156.40)	138.24 (117.10–157.10)	123.90 (105.10–146.70)	**0**.**005**
Serine	102.05 (84.50–122.00)	99.65 (86.80–111.90)	96.90 (80.40–116.30)	93.80 (78.70–108.00)	**0**.**002**
Histidine	83.65 (74.85–94.60)	84.75 (76.40–96.20)	81.70 (71.90–92.90)	77.60 (69.30–87.00)	**<0**.**001**
Lysine	179.65 (148.25–204.45)	179.05 (155.80–206.90)	177.30 (148.60–207.50)	173.50 (145.00–206.30)	0.848
Tryptophan	69.85 (60.25–80.05)	71.30 (60.20–83.60)	71.60 (61.20–83.20)	66.60 (56.90–77.40)	**0**.**034**
Asparagine	46.70 (35.30–57.40)	47.51 (40.70–58.60)	44.70 (33.00–54.70)	44.00 (31.30–55.80)	0.087
Glutamine	516.95 (430.25–578.80)	506.65 (446.50–590.10)	518.07 (439.60–578.90)	512.90 (431.30–598.20)	0.925

*FDR: False Discovery Rate (Adjusted P-value).

The result of the logistic regression on metabolites to discriminate borderline/intermediate/high ASCVD risk patients from low-risk ones is presented in Additional File S5. Patients with high ASCVD risk score were more likely to have an increase in the concentration of 24 acylcarnitines and nine amino acids (*p*-value ≤ 0.041) and were less likely to have an increase in five amino acids than low ASCVD risk group (*p*-value ≤ 0.010). Furthermore, an increase in 18 acylcarnitines and 13 amino acids was more likely to occur in intermediate ASCVD risk patients than in low ASCVD risk patients (*p*-value ≤ 0.044). However, an increase in two amino acids was less likely to occur in intermediate ASCVD risk patients than in low ASCVD risk patients (*p*-value ≤ 0.013). Patients with borderline ASCVD risk score were more likely to have an increase in 24 acylcarnitines and nine amino acids than low ASCVD risk patients (*p*-value ≤ 0.041) and were less likely to have an increase in five amino acids than low ASCVD risk patients (*p*-value ≤ 0.010). The logistic regression analysis was repeated after adjustment for BMI; in the new analysis, the significant *p*-values remained significant, and non-significant ones remained non-significant.

### Metabolite profile and 10-year ASCVD risk

Additional File S6 shows the correlation between the metabolite profile and the 10-year ASCVD risk score. Thirty acylcarnitines and nine amino acids were positively associated with the ASCVD risk score (*p* ≤ 0.034) and four amino acids were inversely related to it (*p* ≤ 0.026). The strongest associations belonged to 3-OH-hexadecanoylcarnitine (C_16_OH, r = 0.279, *p* < 0.001), propionyl carnitine (C_3,_ r = 0.262, *p* < 0.001), leucine (r = 0.256, *p* < 0.001), isovaleryl carnitine (C_5,_ r = 0.251, *p* < 0.001), and 3-OH-isovalerylcarnitine (C_5_OH, r = 0.250, *p* < 0.001). The multiple linear regression on all measured metabolites is summarized in Table 4. Three acylcarnitines (methylmalonyl-/succinyl carnitine [C_4_DC], octenoyl carnitine [C_8:1_], and 3-OH-hexadecanoylcarnitine [C_16_OH]) and 11 amino acids (citrulline, histidine, alanine, threonine, glycine, glutamine, tryptophan, phenylalanine, glutamic acid, arginine, aspartic acid) were concluded as possible predictors of the 10-year ASCVD risk score.

**Table 4:** The multiple linear regression of the metabolites influencing 10-year ASCVD risk score.

Metabolites	Unstandardized coefficients		Standardized coefficients	t	*p*-value
	*β*	Standard error	beta		
(Constant)	−0.265	2.922		−0.091	0.928
C_4_DC	27.122	10.384	0.079	2.612	**0**.**009**
C_8:1_	−5.143	1.886	−0.079	−2.727	**0**.**007**
C_16_OH	426.657	76.198	0.166	5.599	**<0**.**001**
Citrulline	0.281	0.037	0.239	7.515	**<0**.**001**
Histidine	−0.14	0.025	−0.204	−5.503	**<0**.**001**
Alanine	0.015	0.004	0.129	4.232	**<0**.**001**
Threonine	−0.037	0.011	−0.111	−3.37	**0**.**001**
Glycine	−0.014	0.004	−0.098	−3.395	**0**.**001**
Glutamine	0.013	0.003	0.139	3.882	**<0**.**001**
Tryptophane	−0.097	0.022	−0.144	−4.468	**<0**.**001**
Phenylalanine	0.109	0.032	0.114	3.405	**0**.**001**
Glutamic acid	0.089	0.031	0.099	2.901	**0**.**004**
Arginine	−0.038	0.018	−0.065	−2.076	**0**.**038**
Aspartic acid	−0.213	0.106	−0.069	−2.017	**0**.**044**

### Metabolite-derived factors and ASCVD risk

In the factor analysis, the KMO coefficient was 0.874, indicating adequate sampling of data. A *p*-value of <0.001 from Bartlett's sphericity test suggested a statistical difference between the correlation and the identity matrix. Both tests revealed the data to be appropriate for factor analysis.

PCA analysis on the standardized metabolites resulted in 11 factors with an eigenvalue of more than one in the screen plot (Additional File S7). The logistic regression analysis of the borderline/intermediate/high-risk groups compared to the low-risk group is illustrated in Table 5. Patients with high ASCVD risk score were more likely to have an increase in factors 1, 2, 3, 5, 6, 7, 8, and 10 than low ASCVD risk group (*p*-value ≤ 0.009) and were less likely to have an increase in factor nine (*p*-value < 0.001). The intermediate-risk group was more likely to have an increase in factors 1, 3, 5, 6, 7, 8, and 10 than the low-risk group (*p*-value ≤ 0.001) and was less likely to have an increase in factor 9 than the low-risk group (*p*-value = 0.001). Patients with borderline ASCVD risk score were more likely to have an increase in factors 3, 5, 6, 7, and 10 than the low-risk group (*p*-value ≤ 0.009).

**Table 5:** The logistic regression between the factors extracted by PCA and 10-year ASCVD risk score.

Indices	Groups	OR	95% CI	*p*-value*
Factor 1	Borderline-risk	1.021	(0.984–1.059)	0.271
	Intermediate-risk	1.061	(1.034–1.089)	**0**.**000**
	High-risk	1.103	(1.072–1.134)	**0**.**000**
Factor 2	Borderline-risk	1.040	(0.984–1.099)	0.164
	Intermediate-risk	1.011	(0.965–1.060)	0.645
	High-risk	1.063	(1.015–1.112)	**0**.**009**
Factor 3	Borderline-risk	1.082	(1.020–1.147)	**0**.**009**
	Intermediate-risk	1.106	(1.058–1.156)	**0**.**000**
	High-risk	1.074	(1.019–1.132)	**0**.**008**
Factor 4	Borderline-risk	1.033	(0.960–1.112)	0.384
	Intermediate-risk	0.985	(0.930–1.042)	0.593
	High-risk	0.971	(0.907–1.039)	0.395
Factor 5	Borderline-risk	1.146	(1.061–1.238)	**0**.**001**
	Intermediate-risk	1.193	(1.125–1.265)	**0**.**000**
	High-risk	1.205	(1.128–1.287)	**0**.**000**
Factor 6	Borderline-risk	1.195	(1.077–1.327)	**0**.**001**
	Intermediate-risk	1.146	(1.055–1.245)	**0**.**001**
	High-risk	1.229	(1.121–1.348)	**<0**.**001**
Factor 7	Borderline-risk	1.346	(1.121–1.617)	**0**.**001**
	Intermediate-risk	1.294	(1.123–1.491)	**<0**.**001**
	High-risk	1.343	(1.140–1.582)	**<0**.**001**
Factor 8	Borderline-risk	1.184	(1.004–1.396)	0.045
	Intermediate-risk	1.275	(1.127–1.443)	**<0**.**001**
	High-risk	1.188	(1.025–1.377)	**0**.**022**
Factor 9	Borderline-risk	0.922	(0.795–1.070)	0.286
	Intermediate-risk	0.828	(0.737–0.930)	**0**.**001**
	High-risk	0.741	(0.642–0.856)	**<0**.**001**
Factor 10	Borderline-risk	1.473	(1.231–1.762)	**<0**.**001**
	Intermediate-risk	1.534	(1.336–1.762)	**<0**.**001**
	High-risk	1.570	(1.338–1.841)	**<0**.**001**
Factor 11	Borderline-risk	1.101	(0.801–1.513)	0.554
	Intermediate-risk	0.980	(0.770–1.247)	0.868
	High-risk	0.918	(0.690–1.223)	0.560

The reference category is the low-risk group.

* After adjustment for BMI, the significant *p*-values remain significant and non-significant *p*-values remain non-significant.

### The enriched metabolic pathways mediated between study groups

**Figure 2:**
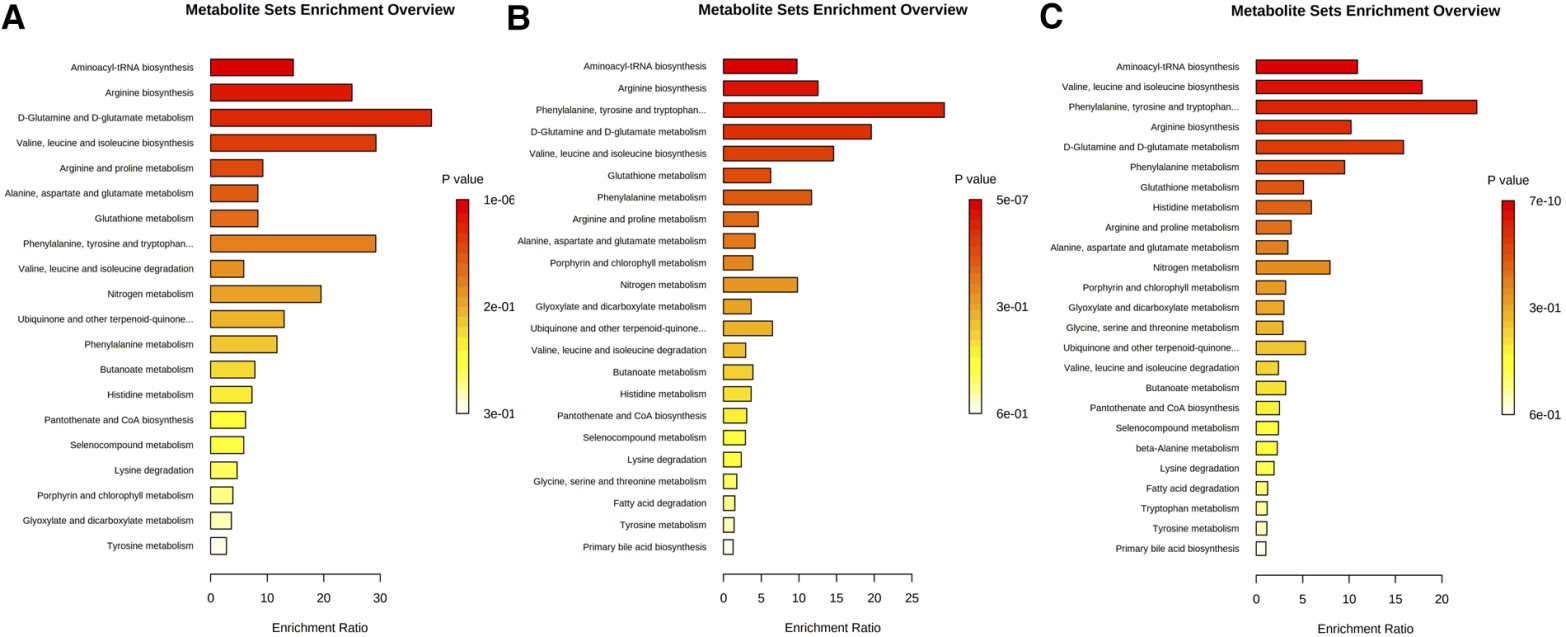
Pathway enrichment analysis between (**A**) low ASCVD risk group and borderline ASCVD risk group, (**B**) low ASCVD risk group and intermediate ASCVD risk group, and (**C**) low ASCVD risk group and high ASCVD risk group. The color of each pathway is based on the *p*-value [-log(p): logarithm of the *p*-value, Fisher's test *p*-value].

**Figure 3:**
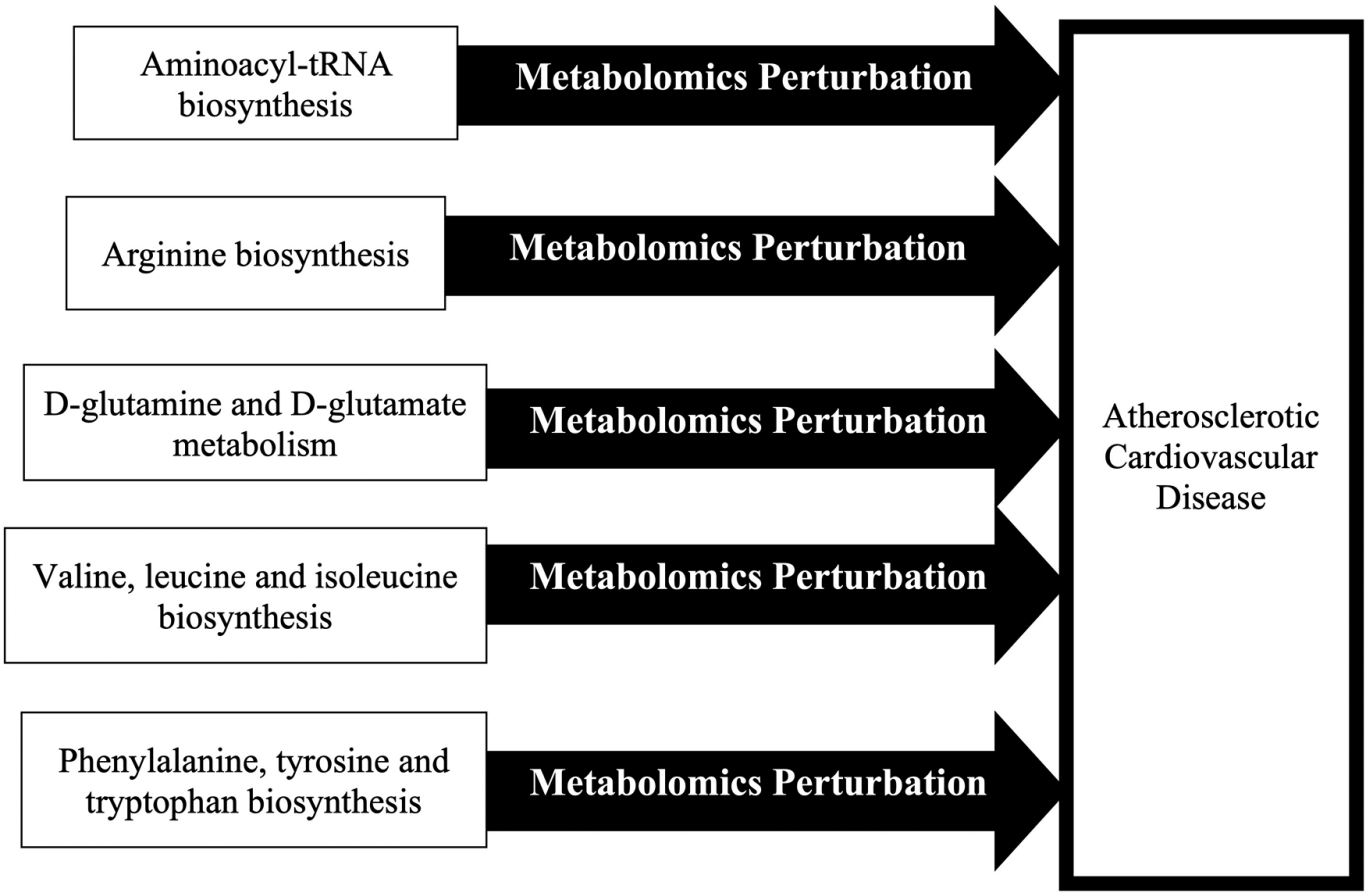
Working model of metabolomic perturbance and ASCVD.

The results of pathway enrichment analysis of altered metabolites in low ASCVD risk group with borderline/intermediate/high ASCVD risk groups are summarized in Additional File S8. The differential metabolites between the low ASCVD risk group and borderline/intermediate/high ASCVD risk groups were mainly associated with the biological processes of “aminoacyl-tRNA biosynthesis”, “arginine biosynthesis”, “D-glutamine and D-glutamate metabolism”, “valine, leucine and isoleucine biosynthesis”, and “phenylalanine, tyrosine, and tryptophan biosynthesis” (Figures 2A–C). The metabolic pathways with the highest enrichment ratio in explaining the differences in metabolic profile between the low ASCVD risk group and the borderline/intermediate/high ASCVD risk groups were “D-glutamine and D-glutamate metabolism”, “phenylalanine, tyrosine, and tryptophan biosynthesis”, and “valine, leucine, and isoleucine biosynthesis”, respectively. The working model of the metabolic perturbance is shown in Figure 3.

## Discussion

It has been discovered that the metabolite profile has a significant correlation with ASCVD risk such as coronary artery disease. Moreover, certain metabolic features can be considered independent and valuable predictors of cardiovascular events with regard to early diagnosis, treatment strategies, and better risk stratification (13). In this study, the 10-year ASCVD risk score was used to classify individuals into four groups: low-, borderline-, intermediate-, and high-risk of ASCVD events. Consequently, their metabolite profile (the plasma concentration of 30 acylcarnitines and 20 amino acids) was measured to determine the association between metabolite profile and the risk of 10-year ASCVD events.

The results of our study indicated a positive correlation between all 30 acylcarnitines, alanine, glutamic acid, leucine, phenylalanine, tyrosine, valine, citrulline, ornithine, and proline and the 10-year ASCVD risk score and a negative correlation of glycine, threonine, serine, and histidine with this score. In accordance with our findings, Würtz P et al. (12) showed a positive correlation between the circulating concentrations of phenylalanine and tyrosine and cardiovascular risk. Ruiz-Canela M et al. (17) in a case-cohort study have shown the direct correlation between higher concentrations of branched-chain amino acids (BCAAs) including leucine and valine, and higher CVD risk. The important role of BCAAs as the predictive markers of cardiovascular events has been also suggested in another study by Hu W et al. (18). Vaarhorst AA et al. (19) reported that blood concentrations of valine, ornithine, and glutamate were associated with coronary heart disease. Shah SH et al. (13) found a considerable association between the plasma levels of glutamate, leucine, isoleucine, citrulline, C_2_, C_8_, C_10:1_, C_14:2_ and CVD risk in the same direction that we found in the current study. Besides, the elevated levels of serum C2 and C8 acylcarnitines are in relation to higher cardiovascular death risk. They could also be correlated with a modest increase in the risk of fatal/nonfatal acute myocardial infarction in patients with stable angina pectoris (20). As shown in Table 6, these findings from the previous studies are in line with our findings.

**Table 6:** Comparisons between results of metabolomic studies and current study.

Metabolomic study	Metabolite alteration in high CVD risk	Results of current study
Würtz P et al. (12)	Higher phenylalanine and tyrosine	Higher phenylalanine and tyrosine
Ruiz-Canela M et al. (17)	Higher leucine and valine	Higher leucine and valine
Hu W et al. (18)	Higher leucine, isoleucine, valine	Higher leucine and valine
Vaarhorst AA et al. (19)	Higher valine, ornithine, and glutamate	Higher valine, ornithine, and glutamic acid
Shah SH et al. (13)	Higher glutamate, leucine, isoleucine, citrulline, C_2_, C_8_, C_10:1_, C_14:2_	Higher glutamic acid, leucine, citrulline, C_2_, C_14.2_
Strand E et al. (20)	Higher C_2_, C_8_, and C_16_	Higher C_2_ and C_16_

This study has also found from multiple linear analysis that C_4_DC, C_8:1_, and C_16_OH, citrulline, histidine, alanine, threonine, glycine, glutamine, tryptophan, phenylalanine, glutamic acid, arginine, and aspartic acid could be considered as new candidate biomarkers for the 10-year ASCVD risk. Based on a study by Hu W et al. (18), there is an independent association between BCAAs and intima-media thickness as a marker of coronary artery disease. Several other studies have also indicated a strong association between acylcarnitines and cardiovascular events (21, 22). In addition, metabolite disturbances can also contribute to the CVD risk factors such as diabetes mellitus as found in many studies (23–27). In this regard, plasma levels of BCAA and aromatic amino acids (AAAs) particularly tyrosine, phenylalanine, and isoleucine can predict both diabetes mellitus and CVD events (28, 29).

Metabolomics can help us identify new intermediate metabolites to better understand the underlying mechanisms responsible for higher 10-year ASCVD risk. For instance, the odds ratio between low- and high/intermediate-risk patients can be explained by certain underlying pathways including aminoacyl-tRNA biosynthesis, valine, leucine, and isoleucine biosynthesis, arginine biosynthesis, phenylalanine, tyrosine, and tryptophan biosynthesis, D-glutamine and D-glutamate metabolism, phenylalanine metabolism, and glutathione metabolism. The role of these underlying mechanisms in the development of cardiovascular events has been stated in previous studies (30–32). For instance, the aminoacyl-tRNA synthetases are found to have an important role in modifying the function of regulatory proteins in different cellular processes. The effects of aminoacyl-tRNA synthetase pathways on coronary arteries, aorta, cardiomyocytes, and fibroblasts and their strong association with angiogenesis and cardiomyopathy have been identified previously, and it suggested the potential role of aminoacyl-tRNA synthetase pathways as therapeutic/diagnostic targets of ASCVDs (30). In addition, the disturbed mechanisms of valine, leucine, isoleucine, and their *α*-keto acids were reported in animals suffering from cardiac events (31, 32). Arginine is another amino acid with an important role in different cellular processes such as protein biosynthesis. Furthermore, it is also involved in vascular tone and endothelial function affecting cardiovascular function (33). Another study has also introduced phenylalanine, tyrosine, and tryptophan biosynthesis (using multi-omics approaches) to be involved in vascular function alterations (34). Phenylalanine metabolism has also been shown to have considerable effects on predicting poor outcomes in critically ill patients presenting with heart failure (35). The pathogenic role of high phenylalanine levels in cardiac aging has also been found (36). On the other hand, glutathione metabolism is also known to have considerable effects on various cell functions during CVD events such as cytokine production and protein synthesis. In this regard, it has been stated that glutathione deficiency can be associated with heart attack and stroke (37). Besides, glutathione metabolism is associated with poor outcomes such as death or re-hospitalization in heart failure patients (38).

Published articles regarding the metabolomics analysis of CVD risk, particularly those on amino acids or acylcarnitines mainly utilize the Framingham risk score for risk estimation of CVD events (13, 18). Despite the effectiveness of the ASCVD risk score and its preference in many cases (39), there are not many articles that have used the ASCVD risk score for that purpose. On the other hand, there have been fewer investigations on acylcarnitines compared with other metabolites like amino acids. The other strength of this study is its large population and broad list of investigated metabolites, which makes its results more valuable. Besides, various acylcarnitines generally showed a stronger association with CVD risk in fasting subjects than in nonfasting subjects (20); so, we measured the concentration of metabolites in fasting plasma. However, this study also has some limitations. The cross-sectional nature of the study prevents us from finding any temporality and actual causality associations between the 10-year ASCVD risk and metabolite profile. This study was conducted on Iranian individuals, which limits the generalization of the results to different populations. Also, the risk of actual ASCVD events according to the metabolic profile was not investigated. Additionally, meaningful differences in the studied patient groups might have affected the metabolite levels. Although these variabilities were adjusted in the risk score estimation equation, their existence is one of the limitations of the present study. Despite all the benefits of metabolic profiling in predictive strategies, its generalizability and reproducibility have to be investigated through comprehensive studies to establish its potential usefulness in the clinical setting (40). Thus, longitudinal studies are needed to confirm the predictive value of the metabolite profile on the ASCVD risk.

## Conclusions

This study identified many amino acids and acylcarnitines as metabolite fingerprints of individuals at higher risk of 10-year ASCVD events. Among them, our results suggest C_4_DC, C_8:1_, C_16_OH, citrulline, histidine, alanine, threonine, glycine, glutamine, tryptophan, phenylalanine, glutamic acid, arginine, and aspartic acid be associated with the 10-year ASCVD risk score and thus could be used as potential predictive biomarkers for the ASCVD events. Furthermore, several metabolic pathways associated with the development of 10-year ASCVD events were identified. Among them, “D-glutamine and D-glutamate metabolism”, “phenylalanine, tyrosine, and tryptophan biosynthesis”, and “valine, leucine, and isoleucine biosynthesis” showed the most influential pathways in this pathogenesis. These findings allow for a better understanding of mechanisms underlying ASCVD events and then were used in diagnostic and therapeutic strategies. However, more studies are still required to confirm this association in larger populations.

**Acylcarnitines name:** Free carnitine (C_0_), Acetyl carnitine (C_2_), Propionyl carnitine (C_3_), Malonyl carnitine (C_3_DC), Butyryl carnitine (C_4_), Methylmalonyl-/succinyl carnitine (C_4_DC), 3-OH-iso-/butyryl carnitine (C_4_OH), Isovaleryl carnitine (C_5_), Tiglylcarnitine (C_5:1_), 3-OH-isovalerylcarnitine (C_5_OH), Glutaryl carnitine (C_5_DC), Hexanoyl carnitine (C_6_), Octanoyl carnitine (C_8_), Octenoyl carnitine (C_8:1_), Decanoyl carnitine (C_10_), Decenoyl carnitine (C_10:1_), Dodecanoyl carnitine (C_12_), Tetradecanoyl carnitine (C_14_), Tetradecenoyl carnitine (C_14:1_), Tetradecadienoyl carnitine (C_14:2_), 3-OH-tetradecanoylcarnitine (C_14_OH), Hexadecanoyl carnitine (C_16_), 3-OH-hexadecanoylcarnitine (C_16_OH), 3-OH-hexadecenoylcarnitine (C_16:1_OH), Hexadecenoyl carnitine (C_16:1_), Octadecanoyl carnitine (C_18_), Octadecenoyl carnitine (C_18:1_), 3-OH-octadecanoyl carnitine (C_18_OH), 3-OH-octadecenoyl carnitine (C_18:1_OH), Octadecadienoyl carnitine (C_18:2_).

## Data Availability

The original contributions presented in the study are included in the article/**Supplementary Material**, further inquiries can be directed to the corresponding author/s.
