# Supplementary material for: Metabolomics profile and 10-year atherosclerotic cardiovascular disease (ASCVD) risk score
Source: Front Cardiovasc Med. 2023 May 3;10:1161761. doi: 10.3389/fcvm.2023.1161761 (PMC10188945; doi:10.3389/fcvm.2023.1161761)
Supplement: Supplementary file 1 [file Datasheet1.docx]

Additional files:

Additional file 1. Workflow diagram of statistical analyses used in the study

Additional file 2. Loading values of Principal Component Analysis (PCA) for the extracted factors.

| Factors | Description | Loadings |
| --- | --- | --- |
| 1 | Long-chain ACs | (C16_1OH * 0.781) + (C18_1OH * 0.765) + (C14 * 0.765) + (C16OH * 0.752) + (C14OH * 0.733) + (C16_1 * 0.717) + (C16 * 0.714) + (C18_1 * 0.713) + (C18 * 0.672) + (C18OH * 0.624) + (C14_2 * 0.377) + (C14_1 * 0.592). |
| 2 | Medium-chain ACs | (C10 * 0.944) + (C8 * 0.936) + (C10_1 * 0.929) + (C6 * 0.76) + (C12 * 0.68) |
| 3 | AAAs and BCAAs | (Tyrosine * 0.797) + (Methionine * 0.753) + (Leucine * 0.74) + (Valine * 0.715) + (Tryptophane * 0.702) + (Phenylalanine * 0.676) |
| 4 | Polar AAs | (Lysine * 0.899) + (Glutamine * 0.888) + (Histidine * 0.714) + (Asparagine * 0.687) |
| 5 | Short-chain ACs | (C5DC * 0.397) + (C5_1 * 0.798) + (C5OH * 0.784) + (C5 * 0.701) + (C3 * 0.532) + (C3DC * 0.424) |
| 6 | Short-chain ACs | (C8_1 * 0.68) + (C2* 0.656) + (C4OH * 0.584) + (C0 * 0.43) + (C4DC* 0.324) |
| 7 | Non-polar AAs | (Alanine * 0.657) + (Proline * 0.61) |
| 8 | Other AAs | (C18_2OH * 0.67) + (Glutamic Acid * 0.522) + (Aspartic Acid * 0.454) |
| 9 | Other AAs | (Glycine * 0.753) + (Serine * 0.676) + (Threonine * 0.353) |
| 10 | Urea cycle AAs | (Ornithine * 0.684) + (Citrulline * 0.67) |
| 11 | Other AAs | (Arginine * 0.633) |

Additional file 3: The concentrations of metabolites in four study groups.


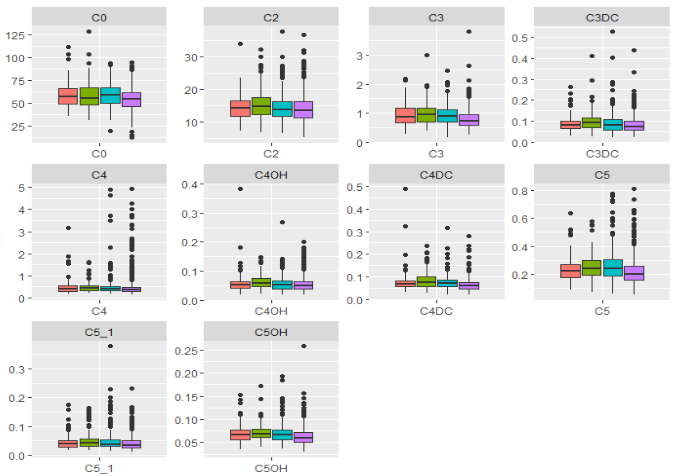


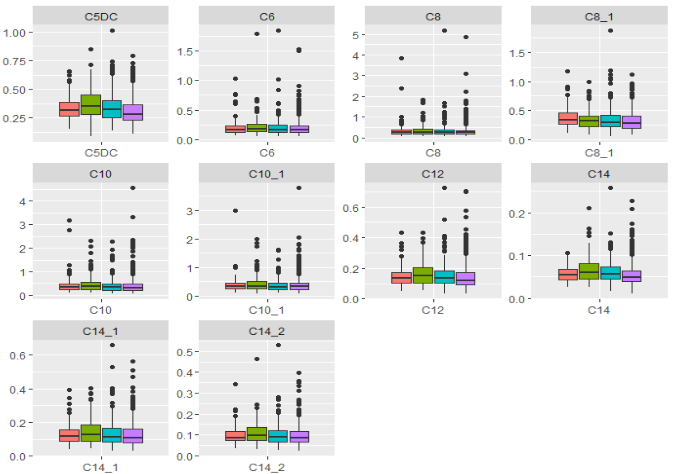


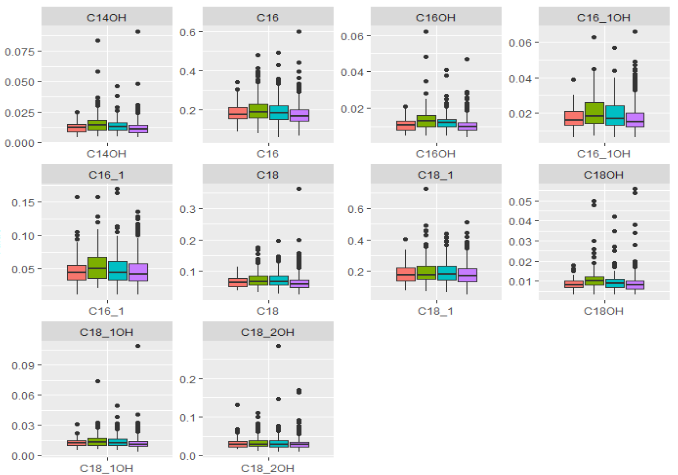


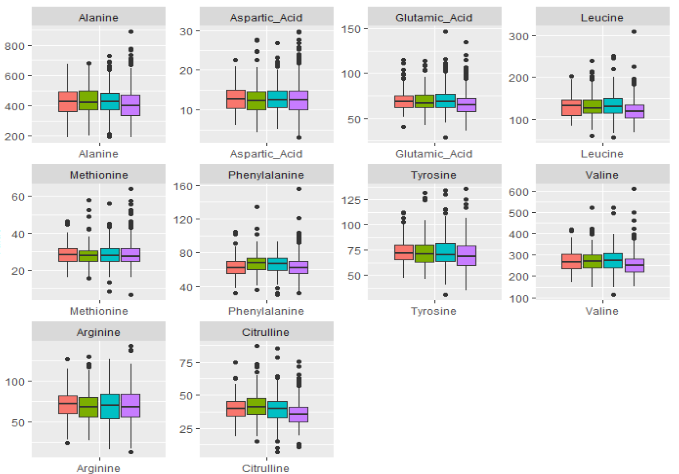


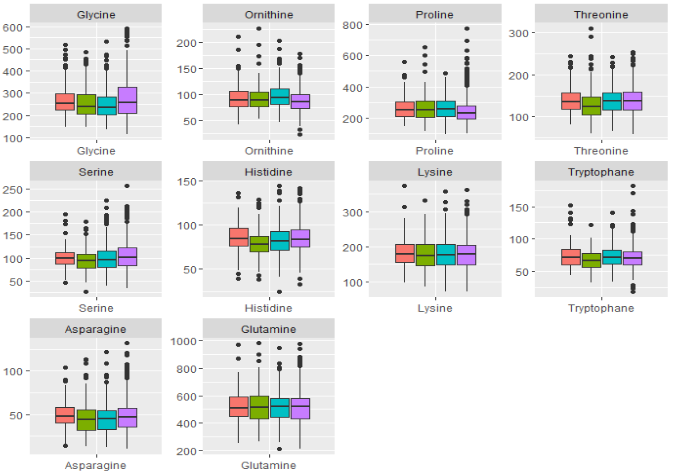


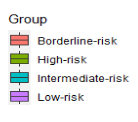


Additional file 4: Significantly altered metabolites among groups’ classification according to sex differences.

| **Metabolites** | **Low-risk** | **Borderline-risk** | **Intermediate-risk** | **High-risk** |
| --- | --- | --- | --- | --- |
| **C0** | **0.012** | 0.844 | 0.972 | 0.875 |
| **C2** | 0.364 | **0.030** | **<0.001** | 0.766 |
| **C3** | **<0.001** | 0.809 | 0.135 | 0.182 |
| **C3DC** | **<0.001** | 0.101 | 0.937 | **0.007** |
| **C4** | 0.147 | 0.135 | **0.002** | 0.914 |
| **C4OH** | 0.860 | **0.035** | **0.007** | 0.724 |
| **C4DC** | **<0.001** | 0.774 | **0.012** | 0.871 |
| **C5** | **<0.001** | 0.087 | **0.002** | **0.002** |
| **C5_1** | 0.627 | 0.586 | 0.818 | 0.766 |
| **C5OH** | **<0.001** | 0.998 | 0.202 | 0.589 |
| **C5DC** | **<0.001** | **0.031** | **0.033** | **<0.001** |
| **C6** | 0.269 | 0.942 | 0.101 | 0.125 |
| **C8** | **<0.001** | 0.993 | 0.138 | 0.073 |
| **C8_1** | 0.955 | **0.005** | **<0.001** | 0.762 |
| **C10** | **<0.001** | 0.580 | 0.081 | 0.059 |
| **C10_1** | **<0.001** | 0.928 | 0.257 | **0.030** |
| **C12** | **<0.001** | 0.472 | 0.407 | 0.052 |
| **C14** | **<0.001** | 0.534 | 0.162 | 0.133 |
| **C14_1** | **0.006** | 0.389 | 0.057 | 0.296 |
| **C14_2** | **<0.001** | 0.887 | 0.873 | **0.008** |
| **C14OH** | **0.002** | 0.403 | **0.004** | 0.515 |
| **C16** | **0.025** | 0.751 | 0.681 | 0.567 |
| **C16OH** | **0.044** | 0.315 | 0.521 | 0.493 |
| **C16_1OH** | **0.003** | 0.293 | 0.227 | 0.354 |
| **C16_1** | 0.072 | **0.003** | **<0.001** | 0.567 |
| **C18** | **<0.001** | 0.047 | 0.272 | 0.123 |
| **C18_1** | 0.706 | 0.285 | 0.494 | 0.687 |
| **C18OH** | **0.009** | 0.932 | 0.152 | 0.727 |
| **C18_1OH** | 0.198 | 0.566 | **0.031** | 0.497 |
| **C18_2OH** | 0.526 | 0.868 | 0.864 | 0.143 |
| **Alanine** | 0.572 | 0.181 | **0.019** | 0.128 |
| **Aspartic Acid** | **0.001** | **0.002** | **0.041** | **0.009** |
| **Glutamic Acid** | **<0.001** | **0.009** | **0.003** | 0.599 |
| **Leucine** | **<0.001** | **<0.001** | **0.001** | **0.030** |
| **Methionine** | **<0.001** | **<0.001** | **<0.001** | **0.001** |
| **Phenylalanine** | **<0.001** | 0.046 | **0.005** | **0.002** |
| **Tyrosine** | **0.004** | 0.809 | **0.039** | 0.109 |
| **Valine** | **<0.001** | **0.007** | 0.120 | 0.420 |
| **Arginine** | 0.225 | **0.012** | **0.014** | **0.006** |
| **Citrulline** | **<0.001** | 0.082 | **<0.001** | **0.006** |
| **Glycine** | 0.053 | **0.018** | **0.021** | **0.022** |
| **Ornithine** | **<0.001** | 0.837 | 0.014 | 0.376 |
| **Proline** | **<0.001** | **0.031** | 0.049 | **0.008** |
| **Threonine** | **0.010** | 0.150 | **0.002** | 0.082 |
| **Serine** | 0.709 | 0.686 | 0.069 | 0.876 |
| **Histidine** | 0.338 | **0.003** | 0.052 | 0.096 |
| **Lysine** | **0.036** | 0.057 | 0.879 | 0.121 |
| **Tryptophane** | **<0.001** | **0.007** | **<0.001** | **0.022** |
| **Asparagine** | **0.010** | 0.055 | **0.004** | **0.005** |
| **Glutamine** | **0.011** | 0.113 | 0.573 | 0.129 |

Additional file 5. The logistic regression analysis on the metabolite profile.

| **Metabolites** | **Groups** | **OR** | **95% CI** | **P-value*** |
| --- | --- | --- | --- | --- |
| C0 | Borderline-risk | 1.370 | (1.117-1.679) | **0.002** |
|  | Intermediate-risk | 1.438 | (1.231-1.680) | **<0.001** |
|  | High-risk | 1.351 | (1.126-1.620) | **0.001** |
| C2 | Borderline-risk | 1.039 | (0.843-1.279) | 0.721 |
|  | Intermediate-risk | 1.103 | (0.946-1.286) | 0.212 |
|  | High-risk | 1.306 | (1.105-1.545) | **0.002** |
| C3 | Borderline-risk | 1.527 | (1.251-1.864) | **<0.001** |
|  | Intermediate-risk | 1.520 | (1.299-1.779) | **<0.001** |
|  | High-risk | 1.630 | (1.367-1.944) | **<0.001** |
| C3DC | Borderline-risk | 1.217 | (0.995-1.489) | 0.056 |
|  | Intermediate-risk | 1.238 | (1.059-1.447) | **0.007** |
|  | High-risk | 1.363 | (1.155-1.609) | **<0.001** |
| C4 | Borderline-risk | 0.967 | (0.777-1.204) | 0.764 |
|  | Intermediate-risk | 1.040 | (0.902-1.199) | 0.586 |
|  | High-risk | 0.952 | (0.780-1.163) | 0.632 |
| C4OH | Borderline-risk | 1.204 | (0.989-1.466) | 0.065 |
|  | Intermediate-risk | 1.135 | (0.966-1.333) | 0.124 |
|  | High-risk | 1.331 | (1.129-1.568) | **0.001** |
| C4DC | Borderline-risk | 1.691 | (1.384-2.067) | **<0.001** |
|  | Intermediate-risk | 1.494 | (1.249-1.788) | **<0.001** |
|  | High-risk | 1.767 | (1.469-2.125) | **<0.001** |
| C5 | Borderline-risk | 1.261 | (1.025-1.552) | **0.028** |
|  | Intermediate-risk | 1.509 | (1.298-1.754) | **<0.001** |
|  | High-risk | 1.384 | (1.159-1.651) | **<0.001** |
| C5:1 | Borderline-risk | 1.137 | (0.921-1.403) | 0.234 |
|  | Intermediate-risk | 1.273 | (1.096-1.477) | **0.002** |
|  | High-risk | 1.260 | (1.063-1.494) | **0.008** |
| C5OH | Borderline-risk | 1.445 | (1.185-1.760) | **<0.001** |
|  | Intermediate-risk | 1.478 | (1.263-1.729) | **<0.001** |
|  | High-risk | 1.493 | (1.252-1.781) | **<0.001** |
| C5DC | Borderline-risk | 1.358 | (1.109-1.663) | **0.003** |
|  | Intermediate-risk | 1.411 | (1.209-1.648) | **<0.001** |
|  | High-risk | 1.644 | (1.382-1.954) | **<0.001** |
| C6 | Borderline-risk | 1.058 | (0.864-1.295) | 0.587 |
|  | Intermediate-risk | 1.069 | (0.917-1.245) | 0.395 |
|  | High-risk | 1.135 | (0.968-1.332) | 0.120 |
| C8 | Borderline-risk | 1.106 | (0.926-1.322) | 0.266 |
|  | Intermediate-risk | 1.045 | (0.890-1.226) | 0.593 |
|  | High-risk | 1.112 | (0.948-1.305) | 0.191 |
| C8:1 | Borderline-risk | 1.323 | (1.105-1.584) | **0.002** |
|  | Intermediate-risk | 1.131 | (0.970-1.318) | 0.116 |
|  | High-risk | 1.099 | (0.916-1.318) | 0.310 |
| C10 | Borderline-risk | 1.077 | (0.888-1.306) | 0.451 |
|  | Intermediate-risk | 0.986 | (0.834-1.164) | 0.864 |
|  | High-risk | 1.157 | (0.990-1.351) | 0.067 |
| C10:1 | Borderline-risk | 1.124 | (0.941-1.344) | 0.198 |
|  | Intermediate-risk | 0.956 | (0.802-1.141) | 0.618 |
|  | High-risk | 1.147 | (0.981-1.340) | 0.085 |
| C12 | Borderline-risk | 1.068 | (0.865-1.319) | 0.542 |
|  | Intermediate-risk | 1.145 | (0.983-1.335) | 0.082 |
|  | High-risk | 1.312 | (1.116-1.542) | **0.001** |
| C14 | Borderline-risk | 1.100 | (0.880-1.375) | 0.404 |
|  | Intermediate-risk | 1.299 | (1.111-1.517) | **0.001** |
|  | High-risk | 1.528 | (1.296-1.801) | **<0.001** |
| C14:1 | Borderline-risk | 0.987 | (0.796-1.225) | 0.909 |
|  | Intermediate-risk | 1.084 | (0.931-1.263) | 0.300 |
|  | High-risk | 1.245 | (1.058-1.465) | **0.008** |
| C14:2 | Borderline-risk | 1.029 | (0.836-1.267) | 0.785 |
|  | Intermediate-risk | 1.062 | (0.911-1.238) | 0.444 |
|  | High-risk | 1.188 | (1.009-1.400) | **0.039** |
| C14OH | Borderline-risk | 1.188 | (0.923-1.529) | 0.181 |
|  | Intermediate-risk | 1.429 | (1.197-1.706) | **<0.001** |
|  | High-risk | 1.789 | (1.482-2.160) | **<0.001** |
| C16 | Borderline-risk | 1.163 | (0.941-1.438) | 0.163 |
|  | Intermediate-risk | 1.338 | (1.147-1.561) | **<0.001** |
|  | High-risk | 1.521 | (1.283-1.802) | **<0.001** |
| C16OH | Borderline-risk | 1.116 | (0.859-1.449) | 0.411 |
|  | Intermediate-risk | 1.665 | (1.400-1.980) | **<0.001** |
|  | High-risk | 1.976 | (1.640-2.380) | **<0.001** |
| C16:1OH | Borderline-risk | 1.052 | (0.844-1.312) | 0.649 |
|  | Intermediate-risk | 1.273 | (1.092-1.484) | **0.002** |
|  | High-risk | 1.535 | (1.302-1.810) | **<0.001** |
| C16:1 | Borderline-risk | 1.019 | (0.820-1.266) | 0.867 |
|  | Intermediate-risk | 1.201 | (1.031-1.398) | **0.018** |
|  | High-risk | 1.410 | (1.196-1.664) | **0.000** |
| C18 | Borderline-risk | 1.225 | (0.975-1.539) | 0.082 |
|  | Intermediate-risk | 1.545 | (1.317-1.812) | **<0.001** |
|  | High-risk | 1.607 | (1.348-1.915) | **<0.001** |
| C18:1 | Borderline-risk | 1.055 | (0.852-1.307) | 0.621 |
|  | Intermediate-risk | 1.214 | (1.042-1.413) | **0.013** |
|  | High-risk | 1.372 | (1.160-1.622) | **<0.001** |
| C18OH | Borderline-risk | 1.079 | (0.825-1.412) | 0.579 |
|  | Intermediate-risk | 1.282 | (1.070-1.535) | **0.007** |
|  | High-risk | 1.606 | (1.342-1.922) | **<0.001** |
| C18:1OH | Borderline-risk | 1.097 | (0.843-1.427) | 0.491 |
|  | Intermediate-risk | 1.358 | (1.137-1.621) | **0.001** |
|  | High-risk | 1.604 | (1.329-1.935) | **<0.001** |
| C18:2OH | Borderline-risk | 1.104 | (0.887-1.375) | 0.374 |
|  | Intermediate-risk | 1.230 | (1.056-1.432) | **0.008** |
|  | High-risk | 1.201 | (1.009-1.429) | **0.040** |
| Alanine | Borderline-risk | 1.302 | (1.064-1.592) | **0.010** |
|  | Intermediate-risk | 1.266 | (1.086-1.477) | **0.003** |
|  | High-risk | 1.354 | (1.132-1.618) | **0.001** |
| Aspartic Acid | Borderline-risk | 1.013 | (0.827-1.240) | 0.901 |
|  | Intermediate-risk | 1.028 | (0.883-1.196) | 0.725 |
|  | High-risk | 0.976 | (0.814-1.170) | 0.792 |
| Glutamic Acid | Borderline-risk | 1.413 | (1.157-1.725) | **0.001** |
|  | Intermediate-risk | 1.508 | (1.293-1.759) | **<0.001** |
|  | High-risk | 1.314 | (1.094-1.577) | **0.003** |
| Leucine | Borderline-risk | 1.517 | (1.237-1.861) | **<0.001** |
|  | Intermediate-risk | 1.645 | (1.405-1.927) | **<0.001** |
|  | High-risk | 1.549 | (1.290-1.859) | **<0.001** |
| Methionine | Borderline-risk | 1.083 | (0.889-1.319) | 0.428 |
|  | Intermediate-risk | 1.047 | (0.900-1.218) | 0.550 |
|  | High-risk | 0.927 | (0.769-1.117) | 0.426 |
| Phenylalanine | Borderline-risk | 1.021 | (0.822-1.268) | 0.854 |
|  | Intermediate-risk | 1.384 | (1.185-1.617) | **<0.001** |
|  | High-risk | 1.512 | (1.267-1.805) | **<0.001** |
| Tyrosine | Borderline-risk | 1.245 | (1.019-1.521) | **0.032** |
|  | Intermediate-risk | 1.302 | (1.119-1.515) | **0.001** |
|  | High-risk | 1.227 | (1.026-1.467) | **0.025** |
| Valine | Borderline-risk | 1.419 | (1.157-1.740) | **0.001** |
|  | Intermediate-risk | 1.554 | (1.329-1.816) | **<0.001** |
|  | High-risk | 1.468 | (1.225-1.760) | **<0.001** |
| Arginine | Borderline-risk | 1.063 | (0.869-1.300) | 0.554 |
|  | Intermediate-risk | 0.987 | (0.847-1.150) | 0.868 |
|  | High-risk | 0.948 | (0.791-1.136) | 0.560 |
| Citrulline | Borderline-risk | 1.599 | (1.301-1.966) | **<0.001** |
|  | Intermediate-risk | 1.556 | (1.324-1.828) | **<0.001** |
|  | High-risk | 1.858 | (1.548-2.230) | **<0.001** |
| Glycine | Borderline-risk | 0.928 | (0.759-1.135) | 0.469 |
|  | Intermediate-risk | 0.734 | (0.621-0.868) | **<0.001** |
|  | High-risk | 0.764 | (0.629-0.929) | **0.007** |
| Ornithine | Borderline-risk | 1.337 | (1.094-1.634) | **0.005** |
|  | Intermediate-risk | 1.473 | (1.265-1.715) | **<0.001** |
|  | High-risk | 1.294 | (1.079-1.551) | **0.005** |
| Proline | Borderline-risk | 1.329 | (1.098-1.608) | **0.004** |
|  | Intermediate-risk | 1.272 | (1.093-1.481) | **0.002** |
|  | High-risk | 1.275 | (1.070-1.519) | **0.007** |
| Threonine | Borderline-risk | 1.057 | (0.867-1.289) | 0.581 |
|  | Intermediate-risk | 0.964 | (0.827-1.123) | 0.638 |
|  | High-risk | 0.759 | (0.625-0.921) | **0.005** |
| Serine | Borderline-risk | 0.840 | (0.680-1.036) | 0.103 |
|  | Intermediate-risk | 0.841 | (0.718-0.984) | **0.031** |
|  | High-risk | 0.675 | (0.551-0.825) | **<0.001** |
| Histidine | Borderline-risk | 1.020 | (0.833-1.249) | 0.848 |
|  | Intermediate-risk | 0.876 | (0.750-1.023) | 0.094 |
|  | High-risk | 0.692 | (0.573-0.835) | **<0.001** |
| Lysine | Borderline-risk | 1.114 | (0.914-1.359) | 0.285 |
|  | Intermediate-risk | 1.026 | (0.881-1.195) | 0.744 |
|  | High-risk | 1.005 | (0.838-1.204) | 0.960 |
| Tryptophane | Borderline-risk | 1.192 | (0.984-1.445) | 0.073 |
|  | Intermediate-risk | 1.099 | (0.946-1.278) | 0.218 |
|  | High-risk | 0.801 | (0.659-0.973) | **0.026** |
| Asparagine | Borderline-risk | 1.100 | (0.905-1.337) | 0.338 |
|  | Intermediate-risk | 0.893 | (0.763-1.046) | 0.160 |
|  | High-risk | 0.946 | (0.788-1.135) | 0.549 |
| Glutamine | Borderline-risk | 1.070 | (0.875-1.309) | 0.509 |
|  | Intermediate-risk | 1.037 | (0.890-1.208) | 0.640 |
|  | High-risk | 1.088 | (0.910-1.300) | 0.356 |

The reference category is: Low risk.

*After adjustment for BMI, the significant p-values remain significant and non-significant p-values remain non-significant.

Additional file 6. The correlation of metabolite profile with 10-years cardiovascular diseases risk based on Framingham risk score.

| **Metabolites** | **Spearman correlation coefficient** | **P-value** | **Metabolites** | **Spearman correlation coefficient** | **P-value** |
| --- | --- | --- | --- | --- | --- |
| C0 | 0.167 | **<0.001** | C18 | 0.236 | **<0.001** |
| C2 | 0.106 | **<0.001** | C18:1 | 0.091 | **0.002** |
| C3 | 0.262 | **<0.001** | C18OH | 0.241 | **<0.001** |
| C3DC | 0.174 | **<0.001** | C18:1OH | 0.182 | **<0.001** |
| C4 | 0.181 | **<0.001** | C18:2OH | 0.152 | **<0.001** |
| C4OH | 0.132 | **<0.001** | Alanine | 0.178 | **<0.001** |
| C4DC | 0.242 | **<0.001** | Aspartic Acid | 0.005 | 0.876 |
| C5 | 0.251 | **<0.001** | Glutamic Acid | 0.192 | **<0.001** |
| C5:1 | 0.135 | **<0.001** | Leucine | 0.256 | **<0.001** |
| C5OH | 0.250 | **<0.001** | Methionine | 0.023 | 0.437 |
| C5DC | 0.240 | **<0.001** | Phenylalanine | 0.19 | **<0.001** |
| C6 | 0.086 | **0.004** | Tyrosine | 0.148 | **<0.001** |
| C8 | 0.086 | **0.004** | Valine | 0.240 | **<0.001** |
| C8:1 | 0.064 | **0.034** | Arginine | -0.008 | 0.798 |
| C10 | 0.089 | **0.003** | Citrulline | 0.247 | **<0.001** |
| C10:1 | 0.067 | **0.025** | Glycine | -0.123 | **<0.001** |
| C12 | 0.163 | **<0.001** | Ornithine | 0.191 | **<0.001** |
| C14 | 0.223 | **<0.001** | Proline | 0.207 | **<0.001** |
| C14:1 | 0.09 | **0.003** | Threonine | -0.067 | **0.026** |
| C14:2 | 0.079 | **0.009** | Serine | -0.164 | **<0.001** |
| C14OH | 0.246 | **<0.001** | Histidine | -0.107 | **<0.001** |
| C16 | 0.201 | **<0.001** | Lysine | 0.022 | 0.471 |
| C16OH | 0.279 | **<0.001** | Tryptophane | 0.024 | 0.419 |
| C16:1OH | 0.185 | **<0.001** | Asparagine | -0.031 | 0.305 |
| C16:1 | 0.115 | **<0.001** | Glutamine | 0.047 | 0.119 |

Additional file 7. Result of Principle Component Analysis (PCA) on the study metabolites.

| Factors | Initial Eigenvalues | | | Extraction Sums of Squared Loadings | | | Rotation Sums of Squared Loadings | | |
| --- | --- | --- | --- | --- | --- | --- | --- | --- | --- |
|  | Total | % Of Variance | Cumulative % | Total | % Of Variance | Cumulative % | Total | % Of Variance | Cumulative % |
| 1 | 11.61 | 23.221 | 23.221 | 11.61 | 23.221 | 23.221 | 7.294 | 14.589 | 14.589 |
| 2 | 6.782 | 13.565 | 36.786 | 6.782 | 13.565 | 36.786 | 5.851 | 11.702 | 26.291 |
| 3 | 3.666 | 7.331 | 44.117 | 3.666 | 7.331 | 44.117 | 4.63 | 9.261 | 35.551 |
| 4 | 2.961 | 5.921 | 50.038 | 2.961 | 5.921 | 50.038 | 3.454 | 6.909 | 42.46 |
| 5 | 2.249 | 4.498 | 54.536 | 2.249 | 4.498 | 54.536 | 3.305 | 6.611 | 49.071 |
| 6 | 1.706 | 3.411 | 57.948 | 1.706 | 3.411 | 57.948 | 2.079 | 4.157 | 53.228 |
| 7 | 1.506 | 3.012 | 60.96 | 1.506 | 3.012 | 60.96 | 1.828 | 3.656 | 56.884 |
| 8 | 1.428 | 2.856 | 63.816 | 1.428 | 2.856 | 63.816 | 1.749 | 3.498 | 60.382 |
| 9 | 1.297 | 2.594 | 66.41 | 1.297 | 2.594 | 66.41 | 1.747 | 3.494 | 63.877 |
| 10 | 1.161 | 2.323 | 68.733 | 1.161 | 2.323 | 68.733 | 1.743 | 3.486 | 67.363 |
| 11 | 1.019 | 2.039 | 70.772 | 1.019 | 2.039 | 70.772 | 1.704 | 3.409 | 70.772 |

Additional file 8. The most highly significant pathways between (A) low-risk vs. borderline-risk, (B) low-risk vs. intermediate-risk, and (C) low-risk vs. high-risk based on metabolic KEGG pathways analysis. The enrichment ratio is calculated based on the observed hits divided by expected hits.

| A | **Metabolite set (Low-risk vs. Borderline-risk)** | **Total** | **Hits** | **Expect** | **Enrichment ratio** | **P value** | **FDR** |
| --- | --- | --- | --- | --- | --- | --- | --- |
| ﻿1 | Aminoacyl-tRNA biosynthesis | 48 | 6 | 0.41 | 14.63 | 1.04E-6 | 8.74E-5 |
| ﻿2 | Arginine biosynthesis | 14 | 3 | 0.12 | 25.00 | 1.68E-4 | 0.00706 |
| ﻿3 | D-Glutamine and D-glutamate metabolism | 6 | 2 | 0.0512 | 39.06 | 9.91E-4 | 0.0278 |
| ﻿4 | Valine, leucine and isoleucine biosynthesis | 8 | 2 | 0.0683 | 29.28 | 0.00183 | 0.0385 |
| ﻿5 | Arginine and proline metabolism | 38 | 3 | 0.325 | 9.23 | 0.00346 | 0.0581 |
| ﻿6 | Alanine, aspartate and glutamate metabolism | 28 | 2 | 0.239 | 8.37 | 0.0225 | 0.27 |
| ﻿7 | Glutathione metabolism | 28 | 2 | 0.239 | 8.37 | 0.0225 | 0.27 |
| ﻿8 | Phenylalanine, tyrosine and tryptophan biosynthesis | 4 | 1 | 0.0342 | 29.24 | 0.0338 | 0.355 |
| ﻿9 | Valine, leucine and isoleucine degradation | 40 | 2 | 0.342 | 5.85 | 0.0438 | 0.408 |
| ﻿10 | Nitrogen metabolism | 6 | 1 | 0.0512 | 19.53 | 0.0502 | 0.422 |
| ﻿11 | Ubiquinone and other terpenoid-quinone biosynthesis | 9 | 1 | 0.0769 | 13.00 | 0.0745 | 0.569 |
| ﻿12 | Phenylalanine metabolism | 10 | 1 | 0.0854 | 11.71 | 0.0824 | 0.577 |
| ﻿13 | Butanoate metabolism | 15 | 1 | 0.128 | 7.81 | 0.121 | 0.773 |
| ﻿14 | Histidine metabolism | 16 | 1 | 0.137 | 7.30 | 0.129 | 0.773 |
| ﻿15 | Pantothenate and CoA biosynthesis | 19 | 1 | 0.162 | 6.17 | 0.151 | 0.832 |
| ﻿16 | Selenocompound metabolism | 20 | 1 | 0.171 | 5.85 | 0.159 | 0.832 |
| ﻿17 | Lysine degradation | 25 | 1 | 0.214 | 4.67 | 0.194 | 0.961 |
| ﻿18 | Porphyrin and chlorophyll metabolism | 30 | 1 | 0.256 | 3.91 | 0.229 | 1.0 |
| ﻿19 | Glyoxylate and dicarboxylate metabolism | 32 | 1 | 0.273 | 3.66 | 0.242 | 1.0 |
| ﻿20 | Tyrosine metabolism | 42 | 1 | 0.359 | 2.79 | 0.306 | 1.0 |
| B | **Metabolite set (Low-risk vs. Intermediate-risk)** | **Total** | **Hits** | **Expect** | **Enrichment ratio** | **P value** | **FDR** |
| ﻿1 | Aminoacyl-tRNA biosynthesis | 48 | 8 | 0.82 | 9.76 | 5.48E-7 | 4.6E-5 |
| ﻿2 | Arginine biosynthesis | 14 | 3 | 0.239 | 12.55 | 0.00142 | 0.0462 |
| ﻿3 | Phenylalanine, tyrosine and tryptophan biosynthesis | 4 | 2 | 0.0683 | 29.28 | 0.00165 | 0.0462 |
| ﻿4 | D-Glutamine and D-glutamate metabolism | 6 | 2 | 0.102 | 19.61 | 0.00404 | 0.0848 |
| ﻿5 | Valine, leucine and isoleucine biosynthesis | 8 | 2 | 0.137 | 14.60 | 0.00738 | 0.124 |
| ﻿6 | Glutathione metabolism | 28 | 3 | 0.478 | 6.28 | 0.0109 | 0.139 |
| ﻿7 | Phenylalanine metabolism | 10 | 2 | 0.171 | 11.70 | 0.0116 | 0.139 |
| ﻿8 | Arginine and proline metabolism | 38 | 3 | 0.649 | 4.62 | 0.0251 | 0.264 |
| ﻿9 | Alanine, aspartate and glutamate metabolism | 28 | 2 | 0.478 | 4.18 | 0.0809 | 0.713 |
| ﻿10 | Porphyrin and chlorophyll metabolism | 30 | 2 | 0.512 | 3.91 | 0.0911 | 0.713 |
| ﻿11 | Nitrogen metabolism | 6 | 1 | 0.102 | 9.80 | 0.0984 | 0.713 |
| ﻿12 | Glyoxylate and dicarboxylate metabolism | 32 | 2 | 0.547 | 3.66 | 0.102 | 0.713 |
| ﻿13 | Ubiquinone and other terpenoid-quinone biosynthesis | 9 | 1 | 0.154 | 6.49 | 0.144 | 0.885 |
| ﻿14 | Valine, leucine and isoleucine degradation | 40 | 2 | 0.683 | 2.93 | 0.147 | 0.885 |
| ﻿15 | Butanoate metabolism | 15 | 1 | 0.256 | 3.91 | 0.229 | 1.0 |
| ﻿16 | Histidine metabolism | 16 | 1 | 0.273 | 3.66 | 0.242 | 1.0 |
| ﻿17 | Pantothenate and CoA biosynthesis | 19 | 1 | 0.325 | 3.08 | 0.281 | 1.0 |
| ﻿18 | Selenocompound metabolism | 20 | 1 | 0.342 | 2.92 | 0.293 | 1.0 |
| ﻿19 | Lysine degradation | 25 | 1 | 0.427 | 2.34 | 0.352 | 1.0 |
| ﻿20 | Glycine, serine and threonine metabolism | 33 | 1 | 0.564 | 1.77 | 0.437 | 1.0 |
| ﻿21 | Fatty acid degradation | 39 | 1 | 0.666 | 1.50 | 0.494 | 1.0 |
| ﻿22 | Tyrosine metabolism | 42 | 1 | 0.717 | 1.39 | 0.52 | 1.0 |
| ﻿23 | Primary bile acid biosynthesis | 46 | 1 | 0.786 | 1.27 | 0.553 | 1.0 |
| C | **Metabolite set (Low-risk vs. High-risk)** | **Total** | **Hits** | **Expect** | **Enrichment ratio** | **P value** | **FDR** |
| ﻿1 | Aminoacyl-tRNA biosynthesis | 48 | 11 | 1.01 | 10.89 | 7.36E-10 | 4.15E-8 |
| ﻿2 | Valine, leucine and isoleucine biosynthesis | 8 | 3 | 0.168 | 17.86 | 4.41E-4 | 0.0168 |
| ﻿3 | Phenylalanine, tyrosine and tryptophan biosynthesis | 4 | 2 | 0.0841 | 23.78 | 0.0025 | 0.0503 |
| ﻿4 | Arginine biosynthesis | 14 | 3 | 0.294 | 10.20 | 0.00263 | 0.0503 |
| ﻿5 | D-Glutamine and D-glutamate metabolism | 6 | 2 | 0.126 | 15.87 | 0.0061 | 0.0962 |
| ﻿6 | Phenylalanine metabolism | 10 | 2 | 0.21 | 9.52 | 0.0174 | 0.213 |
| ﻿7 | Glutathione metabolism | 28 | 3 | 0.589 | 5.09 | 0.0194 | 0.213 |
| ﻿8 | Histidine metabolism | 16 | 2 | 0.336 | 5.95 | 0.0428 | 0.377 |
| ﻿9 | Arginine and proline metabolism | 38 | 3 | 0.799 | 3.75 | 0.0433 | 0.377 |
| ﻿10 | Alanine, aspartate and glutamate metabolism | 28 | 2 | 0.589 | 3.40 | 0.115 | 0.86 |
| ﻿11 | Nitrogen metabolism | 6 | 1 | 0.126 | 7.94 | 0.12 | 0.86 |
| ﻿12 | Porphyrin and chlorophyll metabolism | 30 | 2 | 0.631 | 3.17 | 0.129 | 0.86 |
| ﻿13 | Glyoxylate and dicarboxylate metabolism | 32 | 2 | 0.673 | 2.97 | 0.144 | 0.862 |
| ﻿14 | Glycine, serine and threonine metabolism | 33 | 2 | 0.694 | 2.88 | 0.151 | 0.862 |
| ﻿15 | Ubiquinone and other terpenoid-quinone biosynthesis | 9 | 1 | 0.189 | 5.29 | 0.174 | 0.949 |
| ﻿16 | Valine, leucine and isoleucine degradation | 40 | 2 | 0.841 | 2.38 | 0.204 | 1.0 |
| ﻿17 | Butanoate metabolism | 15 | 1 | 0.315 | 3.17 | 0.274 | 1.0 |
| ﻿18 | Pantothenate and CoA biosynthesis | 19 | 1 | 0.399 | 2.51 | 0.334 | 1.0 |
| ﻿19 | Selenocompound metabolism | 20 | 1 | 0.42 | 2.38 | 0.348 | 1.0 |
| ﻿20 | beta-Alanine metabolism | 21 | 1 | 0.442 | 2.26 | 0.362 | 1.0 |
| ﻿21 | Lysine degradation | 25 | 1 | 0.526 | 1.90 | 0.415 | 1.0 |
| ﻿22 | Fatty acid degradation | 39 | 1 | 0.82 | 1.22 | 0.568 | 1.0 |
| ﻿23 | Tryptophan metabolism | 41 | 1 | 0.862 | 1.16 | 0.586 | 1.0 |
| ﻿24 | Tyrosine metabolism | 42 | 1 | 0.883 | 1.13 | 0.595 | 1.0 |
| ﻿25 | Primary bile acid biosynthesis | 46 | 1 | 0.967 | 1.03 | 0.629 | 1.0 |

Hit: actually matched number from the data

FDR: Adjusted P-value using False Discovery Rate
